# Individualised dosimetry and safety of SIRT for intrahepatic cholangiocarcinoma

**DOI:** 10.1186/s40658-021-00406-2

**Published:** 2021-09-14

**Authors:** Kathy P. Willowson, Enid M. Eslick, Dale L. Bailey

**Affiliations:** 1 Department of Nuclear Medicine, Acute Services Building, Royal North Shore Hospital, St Leonards, NSW 2065 Australia; 2 Institute of Medical Physics, The University of Sydney, Sydney, NSW Australia; 3 Faculty of Medicine and Health, The University of Sydney, Sydney, NSW Australia

**Keywords:** Cholangiocarcinoma, SIRT, Radioembolisation

## Abstract

**Background:**

The aim of this study was to investigate the safety and efficacy of selective internal radiation therapy (SIRT) with ^90^Y resin microspheres for the treatment of Intrahepatic Cholangiocarcinoma (ICC). A total of 23 SIRT procedures from 18 ICC subjects were analysed to determine a lesion-based dose/response relationship with absorbed dose measures from ^90^Y PET and metabolic response as measured on [^18^F]FDG PET. Average absorbed dose (*D*_avg_), minimum dose to 70% of the volume (*D*_70_), volume receiving at least 50 Gy (*V*_50_), biological effective dose (BED) and equivalent uniform dose (EUD), were compared to changes in metabolic volume, maximum standardised uptake value (SUV_max_) and total lesion glycolysis (TLG). Dose to normal liver was assessed with changes in liver uptake rate as measured with [^99m^Tc]mebrofenin scintigraphy for a cohort of 20 subjects with primary liver malignancy (12 ICC, 8 hepatocellular carcinoma (HCC)).

**Results:**

Thirty-four lesions were included in the analysis. A relationship was found between metabolic response and both *D*_avg_ and EUD similar to that seen previously in metastatic colorectal cancer (mCRC), albeit trending towards a lower response plateau. Both dose and SUV coefficient of variation within the lesion (CoV_dose_ and CoV_SUV_), baseline TLG and EUD were found to be mildly significant predictors of response. No strong correlation was seen between normal liver dose and change in [^99m^Tc]mebrofenin liver uptake rate; low baseline uptake rate was not indicative of declining function following SIRT, and no subjects dropped into the ‘poor liver function’ category.

**Conclusions:**

ICC lesions follow a similar dose–response trend as mCRC, however, despite high lesion doses a full metabolic response was rarely seen. The CoV of lesion dose may have a significant bearing on response, and EUD correlated more tightly with metabolic response compared to *D*_avg_. SIRT in primary liver malignancy appears safe in terms of not inducing a clinically significant decline in liver function, and poor baseline uptake rate is not predictive of a reduction in function post SIRT.

**Supplementary Information:**

The online version contains supplementary material available at 10.1186/s40658-021-00406-2.

## Background

Intrahepatic cholangiocarcinoma (ICC) is the second most common form of primary hepatic malignancy, arising from bile duct epithelium in the liver. It is generally associated with a poor prognosis and median survival of only 6.5 months from the time of diagnosis if left untreated, where surgical resection is generally considered the only form of curable treatment [1]. Selective internal radiation therapy (SIRT) is a widely accepted form of treatment for unresectable ICC, with a view to palliation or, in some cases, reducing the bulk of disease in hope of future resection. Similar to hepatocellular carcinoma (HCC), individual lesions can be quite large, which can make targeting the bulk of disease via the lesion vasculature with SIRT challenging.

A number of authors have reported on the safety of SIRT in ICC, which has been shown to be well tolerated, generally results in stabilization of disease, and has significant survival benefits, particularly when stratifying subjects with respect to clinical factors such as multifocal and infiltrative disease [2]. Of note, a recent review article from Zhen et al. [3] concluded that treatment with ^90^Y microspheres was both safe and effective for unresectable ICC with either glass or resin microspheres, despite their differences in specific activity and thus number of spheres injected and dose distribution. The pooled disease control rate measured across nine studies that were reviewed was 77.2%, with no significant differences in both this or overall survival between the two types of microspheres. Improving our understanding of ICC lesion response with respect to absorbed dose delivered during SIRT is key to optimization of the procedure for disease control. A recent study from Manceau et al. [4] used the planning [^99m^Tc]macroaggregated albumin (MAA) study to estimate absorbed dose of glass [^90^Y]microspheres to ICC lesions treated in combination with chemotherapy. Radiological response was evaluated at 3 months in 35 subjects, and a minimal tumour dose for response was reported as 158 Gy. MAA-based dosimetry was also used in conjunction with [^99m^Tc]Sulphur colloid (TcSC) to establish absorbed dose estimates in tumour and in functional liver in a study by Lam et al. [5]. Radiographic responses at 3 and 6 months demonstrated a correlation with tumour absorbed dose, as did overall survival, and the absorbed dose in functional liver was reported to significantly correlate with induced toxicity and with radioembolisation induced liver disease (REILD). Of the 18 ICC subjects included in the study, the median calculated absorbed dose to tumour and functional liver was 35 Gy and 25 Gy, respectively. The median dose for responders at 6 months (across all pathologies) was 60 Gy. A more recent study from Levillain et al. [6] demonstrated that response of ICC to SIRT with resin microspheres strongly depends on tumour dose, highlighting the importance of personalized dosing regimens in SIRT therapy for improved outcome, which resulted in a significant improvement of median overall survival in ICC subjects treated with the partition model *vs* the body surface area model. Whilst radiation dose estimates were again based on [^99m^T]MAA, this article highlights the interest of SIRT in ICC and also the role that personalised dosimetry has to play in optimization of treatment through improved prescribed activity values.

Whilst MAA predictive dosimetry has been presented frequently in the literature, its accuracy and validity has been the topic of much debate for a multitude of reasons, including variations in catheter placement between planning and treatment procedures, changes in vascular supply and targeting if the procedures are not performed within a narrow time window, and differences in particle size and shape and the resulting flow dynamics under injection. Recognition of these factors and awareness of such factors plays a vital role in the correct use of MAA for SIRT planning [7]. A far more robust approach to post-treatment SIRT dosimetry comes from ^90^Y PET/CT, which was first introduced in 2010 [8] and has been extensively used in dosimetry studies of various pathologies targeted with SIRT, particularly metastatic colorectal cancer (mCRC) [9–11]. A recent article from Kafrouni et al. [12] demonstrated a high dependence on catheter position in the reproducibility of dose estimates between MAA SPECT and ^90^Y PET when treating HCC with glass microspheres. However, when positioning was reproduced accurately, such discrepancies were minimized and there was good correlation between both the predicted and true absorbed dose estimates in tumour and normal liver. To our knowledge, there are no studies demonstrating true measured absorbed dose from SIRT using ^90^Y PET/CT specifically in ICC.

The importance of dosimetry for treatment guidance and prediction of response has been highlighted in the recently published international recommendations on personalized SIRT with resin microspheres [13]. The document is a move towards optimization of SIRT and consistency in treatment approach and strategy based on recommendations that have been formulated by an international expert committee, and includes guidance on target doses to various pathologies being treated with SIRT, including ICC. However, the paper does recognize that there are no published reports of the absorbed dose threshold associated with tumour control in this cohort, and thus evidence is still needed to better inform such treatments.

The aim of this study was to use state-of-the-art software tools to generate dose metrics from quantitative ^90^Y PET/CT acquired post-SIRT. Dose-volume histograms (DVHs), derived through dose kernel convolution (DKC), have been investigated and compared to metabolic lesion response as measured with [^18^F]FDG PET/CT to investigate the dose–response relationship for ICC lesions treated with ^90^Y resin microspheres. A further cohort of both ICC and HCC subjects has also been investigated for safety and tolerance of normal liver absorbed dose, with a view to dose escalation in the future for improved lesion response. It should be noted that the dose response relationship derived from this study is specific to resin microspheres, and would be expected to differ for glass microspheres due to the change in specific activity per sphere, and so distribution of dose and the resulting biological effect.

## Methods

All procedures followed were in accordance with the Helsinki Declaration of 1975 (revised in 2000), and all subjects gave informed consent for clinical data to be used for research purposes. All PET/CT data were acquired on a Siemens Biograph mCT with an axial field of view of 24.6 cm (Siemens Healthineers®). ^90^Y data for post hoc dosimetry measurement were acquired as two bed positions centered over the liver, of ten minutes duration each, and were reconstructed with 1 iteration and 21 subsets (OSEM) using an all pass filter, time of flight and point spread function resolution recovery [14]. Acquisitions of [^18^F]FDG PET/CT data followed our standard in-house protocol and were either acquired as a whole body study (2.5 min per bed position, vertex to mid-thigh) approx. 60 min after the injection of 250–300 MBq FDG or as a limited field of view over the liver for planning/response purposes only (6 min per bed, low dose (100 MBq FDG) study), both of which were reconstructed with 3 iterations and 21 subsets including corrections for time of flight and resolution recovery. All therapies were performed with SIR-Spheres resin microspheres, and a personalized approach to dose prescription was used. This consisted of a myriad of factors generally discussed within a multidisciplinary team setting between the nuclear medicine physicist, the nuclear medicine physician and the interventional radiologist, with occasional input from the referring physician where necessary. The Sirtex SMAC (SIR-Spheres Microspheres Activity Calculator) worksheet was used, allowing for the modified BSA method and the partition method to be considered (where appropriate, depending largely on selective or whole liver treatment plans), with heavy guidance from MAA-based predictive dosimetry. Appropriate normal liver dose from predictive dosimetry was also considered in light of baseline dynamic [^99m^Tc]mebrofenin scintigraphy results and standard liver function tests.

Eighteen subjects with ICC were identified retrospectively, all of which had received SIRT with resin microspheres (Sirtex®) between the years 2013–2020, and five of which had received repeat treatment. Twelve of these subjects received a whole liver treatment, one subject received sequential lobar treatment, and five subjects received selective treatments. Table 1 describes the cohort details. All subjects followed the standard protocol for treatment at our site including a baseline [^18^F]FDG PET/CT (approximately 2 weeks prior to treatment), a [^99m^Tc]MAA shunt study including SPECT/CT of the liver, a ^90^Y PET/CT acquired within 24 h after treatment, and a follow-up [^18^F]FDG PET/CT (approximately 8 weeks after treatment).

**Table 1:** Characteristics of ICC patient (*N* = 28), treatment (*N* = 23) and lesion (*N* = 34) population at baseline

Characteristic	*N* (%) unless indicated otherwise
Age in years, median (range)	67 (42–81)
*Sex*	
Male	8 (44%)
Female	10 (56%)
BMI, median (range)	27 (21–35)
Hepatic tumour burden (%)^a^, median (range)	18 (1–42)
Extrahepatic metastases^b^	10 (56%)
*Prior therapies*	
Chemotherapy^b^	13 (72%)
Hepatic resection	2 (11%)
TACE^c^ to liver	3 (17%)
Radiotherapy	2 (11%)
Cirrhosis present	1 (6%)
Treatment to whole liver	12 (67%)
Prescribed amount of ^90^Y for treatment in GBq, median (range)	1.5 (0.6–3.0)
Time to follow-up FDG PET/CT in days, median (range)	51 (42–79)
Lesion volume (cc), median (range)	143 (11–1272)
Lesion SUVmax, median (range)	11.5 (5.0–26.4)
Lesion TLG, median (range)	598 (54–8007)
Overall survival in months, median (range)^d^	6.5 (4.4–19.9)

^a^As measured from FDG PET

^b^Including cisplatin, carboplatin and gemcitabine

^c^Transarterial chemoemnolisation

^d^*N* = 20 (8 patients had no deceased records)

All data were deformably co-registered to a baseline contrast-enhanced CT (CECT) for segmentation of whole liver volume. For each study, up to four lesions with highest uptake were identified on the baseline [^18^F]FDG PET/CT for analysis. Lesions were segmented into volumes of interest (VOIs) based on a threshold on the [^18^F]FDG PET (initially set at 30% of the maximum and adjusted by the user if not appropriate) and metrics of maximum and mean standardized uptake value (SUV_max_, SUV_mean_), VOI volume, total lesion glycolysis (TLG) (defined as the product of VOI volume and SUV_mean_ with units of *g* (*g*/cc × cc)), and coefficient of variation (CoV) of SUV with the VOI (CoV_SUV_) were derived. For each segmented lesion, the average absorbed dose (*D*_avg_), minimum dose to 70% of the volume (*D*_70_) and volume receiving at least 50 Gy (*V*_50_) were derived from dose maps and DVHs generated from the co-registered quantitative ^90^Y PET via DKC. The CoV of absorbed dose within each lesion was also identified (CoV_dose_) to measure the heterogeneity of dose across the volume, which is often considerably large in the ICC cohort. Subtraction of the total tumour burden within the liver defined on FDG PET (based on a threshold applied across the liver volume) from the whole liver volume defined on CECT was used to identify the non-tumour compartment of the liver. Deformable registration was repeated with the follow-up [^18^F]FDG PET/CT data. The same lesions were identified at follow-up and contours redefined through thresholding. Identical FDG-based metrics were derived on the follow-up data, and changes in lesion parameters were compared to absorbed dose values.

Absorbed dose measures across lesions were also extended to estimates of biological effective dose (BED), based on Eq. 1, assuming parameters *T*_eff_ = 64.2 h; *T*_rep_ = 1.5 h; and *α*/*β* = 10 Gy [15]:

1 $${\text{BED}} = 1 + \frac{{D_{{{\text{avg}}}} \times T_{{{\text{rep}}}} }}{{\left( {T_{{{\text{rep}}}} + T_{{{\text{eff}}}} } \right) \times {\raise0.7ex\hbox{$\alpha $} \!\mathord{\left/ {\vphantom {\alpha \beta }}\right.\kern-\nulldelimiterspace} \!\lower0.7ex\hbox{$\beta $}}}}$$

To further explore the effects of dose heterogeneity, the dose modelling was extended to calculation of the equivalent uniform dose (EUD) via Eq. (2), using an ‘apparent’ alpha parameter (clinically derived radiosensitivity) reported for HCC of 0.038 Gy^−1^ [16]; the sum is over each of the lesion voxels *i*, *D*_*i*_ is the absorbed dose measured in voxel *i*, and *N*_vox_ is the total number of voxels in the lesion contour:

2 $${\text{EUD}} = \frac{ - 1}{\alpha }\ln \left( {\frac{{\mathop \sum \nolimits_{i} e^{{ - \alpha \times D_{i} }} }}{{N_{{{\text{vox}}}} }}} \right)$$

An additional cohort of 19 subjects with primary liver malignancy consisting of 7 HCC and 12 ICC (taken from the original cohort of 18) pathologies were also identified as SIRT subjects having had both baseline and follow-up [^99m^Tc]mebrofenin scintigraphy to assess total liver uptake rate as a surrogate of liver function (performed on the same day as [^18^F]FDG PET/CT). Ten of these subjects received whole liver treatment, one subject received sequential lobar treatment, and 8 subjects received selective treatment. One subject had pre-existing cirrhosis at the time of SIRT. All subjects satisfied the safety criteria with respect to pre-treatment blood-based liver function laboratory results and satisfactory mebrofenin function, and all subjects were prescribed the amount of activity in the treatment following the personalized approach described above. These data were used to assess safety of SIRT with resin microspheres and look for any damaging effects of absorbed dose in normal liver on liver function, defined as a clinically significant drop in liver uptake rate (at least 20%). These data were acquired as dynamic planar studies, each of 6 min in duration, divided into 10 s frames (see [17] for more details on analysis). Baseline liver uptake rate was compared to baseline serum bilirubin measures, and changes in liver uptake rate were compared to baseline values, tumour burden and normal liver *D*_avg_ for possible correlation.

All image analysis, including co-registration, dose map and DVH generation, lesion definition and volume statistics, was performed using MIM Software (MIM, Cleveland OH, USA). Statistical analyses were performed using IBM SPSS Statistics (version 25, SPSS Inc.). Significance of prognostic factors was tested using univariate binary logistic regression with a 95% confidence interval (CI) to predict a significant metabolic response (defined as a reduction in TLG of at least 50% [10, 11]). Those parameters with a *p* value of 0.2 or less were retained in the multivariate backward stepwise likelihood ratio regression analysis, excluding parameters with high collinearity, verified by consideration of both clinical factors and correlation coefficients, to identify significant predictors of response.

## Results

A total of 23 SIRT procedures (13 females and 10 males) and 34 lesions were analysed. The mean injected radioactivity of ^90^Y for treatment was 1.62 ± 0.55 GBq (range: 0.6–3.0 GBq). The mean age at treatment was 71 years (range: 42–81). Of the five subjects that had repeat treatments, two of these received treatment to different segments of the liver than from the original treatment. The other three subjects received treatment to the same segment, however due to the extensive spread and morphing of disease, much of the second treatment did not overlap with the initial target. In addition, these subjects had at least 6 months between treatments and for this reason cumulative dose was not considered. Additional file 1: Figure S1 shows an example of the analysis and display software, including baseline and follow-up [^18^F]FDG PET/CT with lesion definition, ^90^Y dose map, and DVH for the lesion contour.

Of the 34 lesions analysed 38% had a significant metabolic response (reduction in TLG of at least 50%). Only 6% had a significant increase in lesion TLG (increase in TLG of at least 50%), or significant progressive metabolic disease. The remaining 56% had stable metabolic disease at follow-up (a change in TLG of less than ± 50%).

**Fig. 1:**
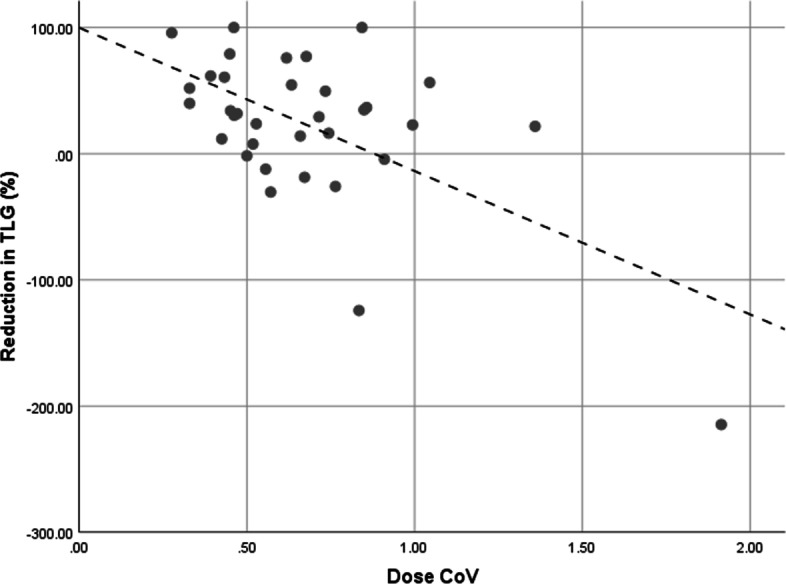
Linear relationship between CoV_dose_ and lesion response (*R*^2^ = 0.48)

Table 2 shows the p-values for the univariate binary logistic regression analysis. None of the variables reached a significance of *p* < 0.05. Baseline TLG, baseline volume, EUD, CoV_dose,_ and CoV_SUV_ demonstrated a *p* value < 0.2. Upon multivariate backward stepwise likelihood ratio regression analysis including these five parameters only two parameters were deemed to be meaningful predictors of a significant metabolic response: Baseline TLG (*p* = 0.138) and CoV_dose_ (*p* = 0.079). Figure 1 represents the relationship between lesion response and CoV_dose_.

**Table 2:** Significance of parameters tested in the univariate binary logistic regression analysis when investigating prognostic factors for a significant reduction in lesion TLG following SIRT

Parameter	***p*** value
Baseline TLG^a^	0.116
Baseline SUV_max_^b^	0.364
Baseline volume (cc)	0.194
*D*_avg_^c^	0.312
*D*_70_^d^	0.606
*V*_50_^e^	0.549
BED	0.613
EUD	0.177
CoV_dose_^f^	0.064
CoV_SUV_^g^	0.176

^a^Total lesion glycolysis

^b^Standardised uptake value (maximum)

^c^Average dose

^d^Minimum dose to 70% of volume

^e^Volume receiving at least 50 Gy

^f^Coefficient of variation of absorbed dose

^g^Coefficient of variation of SUV

Table 3 represents the mean values for each parameter when considering lesions that had a significant metabolic response (*N* = 13) and those that did not (*N* = 21), as well as the significance of each parameter when running an independent samples t-test with a 95% confidence interval. Similarly, when such an analysis was extended to volume reduction as the measure of response (50% reduction being deemed significant in light of potential resection after SIRT), both baseline TLG and CoV_dose_ were again the only parameters of any significance (*p* = 0.138 and *p* = 0.079, respectively).

**Table 3:** Mean of responding and non-responding lesions, where a reduction in TLG of at least 50% was chosen to represent a significant metabolic response

Parameter	Mean value		***p*** value
	Significant response	Non-significant response	
Baseline TLG^a^	932 ± 1522	2152 ± 2302	0.101
Baseline SUV_max_^b^	11.4 ± 3.8	12.3 ± 5.1	0.336
Baseline volume (cc)	182 ± 264	333 ± 346	0.162
D_avg_^c^	74 ± 32	61 ± 38	0.299
D_70_^d^	42 ± 16	37 ± 31	0.617
V_50_^e^	54 ± 26	48 ± 30	0.548
BED	85 ± 44	75 ± 56	0.604
EUD	49 ± 16	39 ± 22	0.146
CoV_dose_^f^	0.542 ± 0.198	0.758 ± 0.356	0.030
CoV_SUV_^g^	0.274 ± 0.103	0.266 ± 0.069	0.806

^a^Total lesion glycolysis

^b^Standardised uptake value (maximum)

^c^Average dose

^d^Minimum dose to 70% of volume

^e^Volume receiving at least 50 Gy

^f^Coefficient of variation of absorbed dose

^g^Coefficient of variation of SUV

**Fig. 2:**
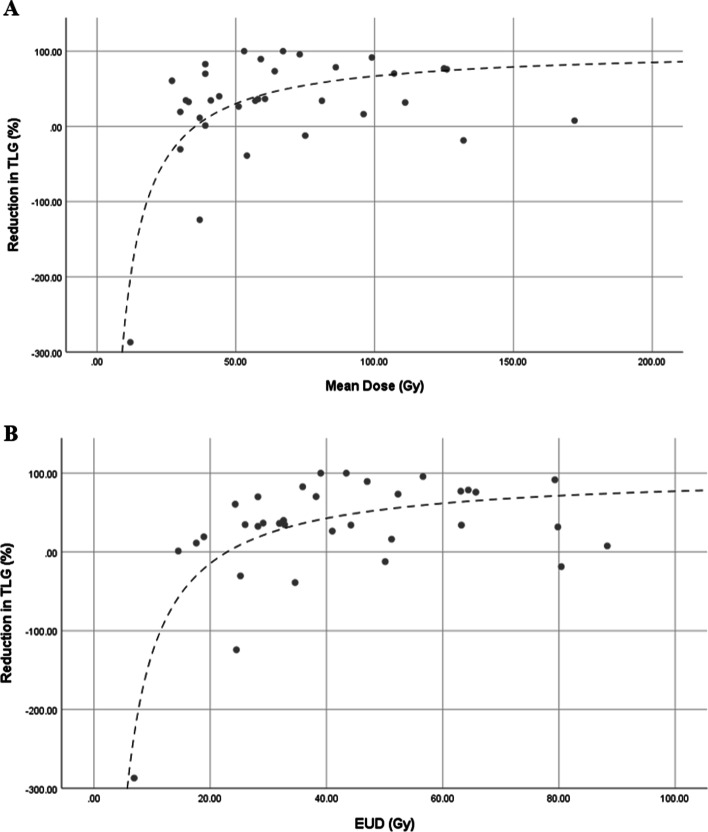
The relationship between average dose (**a**) and equivalent uniform dose (**b**) and lesion response, as measured by reduction in TLG. The line of fit in **a** is represented by the equation *y* = 103.4–3656.9/*x*, with a *R*^2^ value of 0.49; and for (B) *y* = 104.2–3642.7/*x*, with a *R*^2^ value of 0.57

Figure 2a demonstrates the nonlinear relationship between *D*_avg_ and response, which in this case was modelled with an inverse fit using equation *y* = 103.4–3656.9/*x,* with a *R*^2^ value of 0.49. This relationship was extended to EUD (Fig. 2b) to further take into account dose heterogeneity, which resulted in an *R*^2^ value to 0.57. Response as a function of BED yielded results remarkably similar to the use of *D*_avg_, and as such have not been displayed. It should also be noted that using change in lesion volume as a measure of response, if considering the goal of SIRT to shrink lesions to a resectable size, the relationship with *D*_avg_ and CoV_dose_ demonstrated a similar trend.

Across the ICC/HCC cohort, the mean *D*_avg_ and *D*_70_ to healthy liver for a single procedure was 21 ± 8 Gy and 7 ± 5 Gy, respectively (range of *D*_avg_: 4–37 Gy). The largest cumulative *D*_avg_ to healthy liver in a subject who received two procedures was 58 Gy. No incidents of REILD were reported.

**Fig. 3:**
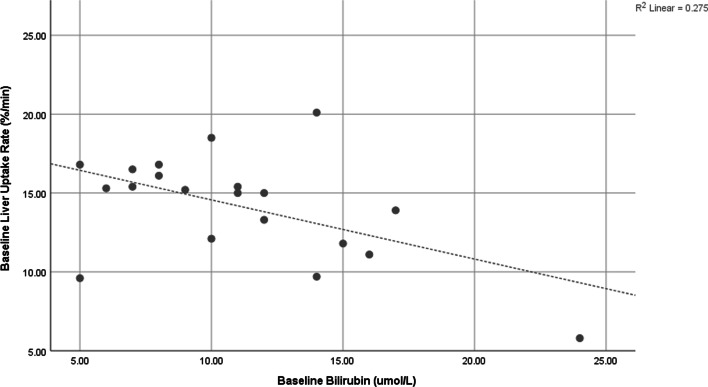
Comparison of baseline Bilirubin measures in blood and baseline global liver uptake rate as measured by ^99m^Tc-mebrofenin dynamic scintigraphy

Whilst 6 of the 20 subjects had a significant decrease in liver uptake rate calculated from [^99m^Tc]mebrofenin dynamic data (i.e., at least a 20% reduction) following SIRT, no subjects fell into the ‘poor liver function’ category after SIRT as defined by a normal liver uptake rate of at least 8.7%/min [18]. There was no significant difference between the means in either baseline liver uptake rate, baseline bilirubin or tumour burden for those subjects that had a significant reduction in liver function and those that did not. The relationship between baseline bilirubin and baseline liver uptake rate can be seen in Fig. 3. Additional file 4: Table S4 shows the p-value for the univariate binary logistic regression analysis when treating a significant drop in liver uptake rate as the test statistic. When considering multivariate backward stepwise likelihood ratio regression, baseline liver uptake rate and *D*_avg_ to healthy liver demonstrated p-values of 0.060 and 0.100, respectively.

**Fig. 4:**
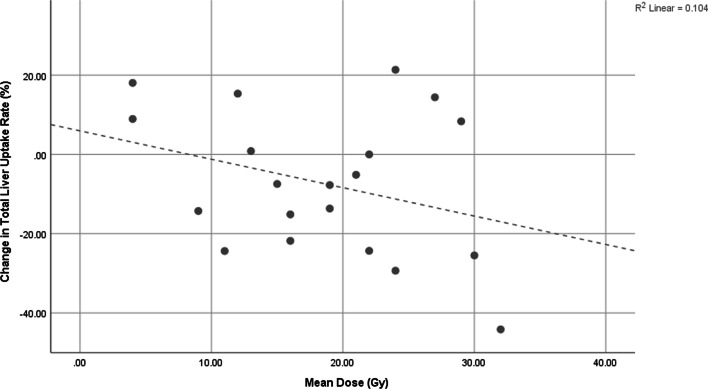
Change in liver function, as measured by the global liver uptake rate (%/min) from ^99m^Tc-mebrofenin dynamic scintigraphy, with the mean absorbed dose to healthy liver parenchyma following SIRT (*R*^2^ of linear fit is 0.104)

Figure 4 demonstrates the lack of correlation between *D*_avg_ in healthy liver and change in liver uptake rate. When considering *D*_70_ this did not change. Additional file 2: Figure S2 demonstrates the negative linear correlation between baseline liver uptake rate and change in liver uptake rate following SIRT. These results suggests that a low liver uptake rate at baseline alone should not preclude ICC/HCC subjects from receiving SIRT as this does not appear to be prognostic for a decline in liver function post-SIRT but instead these subjects may well experience an improvement in function.

## Discussion

In this cohort of ICC subjects, SIRT appears to be a well-tolerated form of therapy achieving high tumour control as demonstrated by the fact that the majority of subjects (94%) had either stable disease or significant metabolic response in their treated lesions following treatment.

Statistical analysis did not identify significant prognostic parameters (*p* < 0.05) when predicting response, however, this may be a reflection of sample size and other clinical factors having a bearing on treatment outcome such as additional pathological sub-categories, mutation status of disease, and prior therapies. However, the data do suggest that baseline TLG, EUD, and CoV of both SUV and dose may act as significant predictors of response to treatment. As demonstrated in Fig. 1, a smaller CoV_dose_ (i.e., more homogenous targeting) was associated with an improved response, and all lesions with a CoV_dose_ below the cut-off value of 0.542 (see Table 3) experienced a reduction in TLG. Given the often large volumes of disease being treated, and the heterogeneity of absorbed dose seen across this volume, this is not an unexpected result. This may be supported by the higher prognostic power of EUD versus *D*_avg_ when correlating with response. By virtue of the aberrant blood supply to ICC lesions the dose does not merely deposit around the periphery of disease, a behavior often associated with large lesions receiving SIRT, but appears to distribute throughout the lesion. However, the dose is not uniform across the lesion volume. It appears that a more uniform dose results in an improved response to SIRT, minimizing pockets of spared disease that can readily progress despite radiation treatment. In Fig. 2 the response curve follows a very similar trend to that previously reported for mCRC with SIRT [10], however, the curve does not tend to plateau at a complete metabolic response. These data suggest a lower plateau, perhaps in the vicinity of 80% reduction in TLG. This is despite the fact that high average doses are being achieved and is again possibly indicative of CoV_dose_ playing a role and the importance of parameters such as EUD to capture this. It is worth noting that the cohort of lesions in this study tended to have relatively low CoV_dose_ values when compared, for example, to previously published mCRC data [10]; in this cohort the mean CoV_dose_ associated with non-responders (0.758) was less than the relevant threshold derived for mCRC lesions of 0.79. We must also recognize that the limitations of the PET spatial resolution which prevents the demonstration of microscopic dose heterogeneity due to non-uniform microsphere deposition being taken into account in such a calculation.

Whilst EUD tended to show a tighter fit of data around the trend line when mapping dose–response when compared to *D*_avg_, the parallel trend between the two parameters is in keeping with reports of both EUD and *D*_avg_ being comparable when estimating tumour control probabilities for SIRT with glass microspheres [19]. Also of interest when comparing to recent literature that has considered both *D*_avg_ and EUD (using the same apparent alpha as this study), the linear fit between the two parameters across this cohort returned a slope of 0.62 compared to that reported by d’Abadie et al. of 0.74 [see Additional file 3: Figure S3] [16]. 

Considering dose metrics, the data are not necessarily straight forward to interpret. We find an example of a lesion receiving a physical dose of 172 Gy, a mean BED of nearly 250 Gy and an EUD of 88 Gy, yet still not achieving a good metabolic response. The CoV_dose_ in this case was 0.518—below the threshold indicated in Table 3, and the baseline TLG was below the population mean (353 g), suggesting other factors that have not been considered in the analysis may be involved. Likewise, there are examples of subjects with multiple lesions achieving a mixed metabolic response that does not always correlate with dose or EUD. The treatment of ICC with EBRT has been reported as having favorable outcome for BEDs greater than 80.5 Gy [20]. Comparing with our results from ^90^Y, it appears the major differences between the delivery of the two doses is through uniformity and also the effects of dose rate—which is inherently and significantly less for ^90^Y than for EBRT. The literature has suggested a radiation biological effectiveness of physical ^90^Y doses of approximately 0.4–0.6 when compared to EBRT [21], meaning a lesion receiving a physical absorbed dose of 172 Gy (as above) should in theory be scaled to a range of 69–103 Gy (or a BED of 72–136 Gy)—still comparable or in excess of EBRT thresholds. As such, this may suggest the non-uniformity is the biggest factor contributing to a less than expected response of ICC to SIRT in some instances.

Considering the recently published guidelines suggesting a mean absorbed dose of 100–120 Gy for ICC lesions targeted with SIRT with resin microspheres [13], if we consider the seven lesions that did achieve this threshold four of these demonstrated a significant response to treatment and the remaining three demonstrated stable disease with no lesions in this category demonstrating progressive disease. However, within this cohort there was no clear pattern as to those lesions that responded significantly and those that did not when considering all other metrics that had been examined.

It should also be recognized that the BED and EUD models for ^90^Y may still be in need of improvement in terms of modelling α/β ratios in the linear quadratic model specifically for ICC cells with ^90^Y, as well as incorporating the full spectrum of ^90^Y emissions into the modelling of dose deposition. Such work is an area of interest and growth in radionuclide therapy, where early results demonstrate a significant need to increase our understanding of the radiobiology [22].

The literature has also demonstrated much promise for immunotherapy administered in combination with radiation, including SIRT, to enhance response to treatment which could help address the issue of non-uniformity of dose deposition across large disease volumes as often seen in ICC (“chemo-sensitising radiaton”). A recent study in HCC [23] demonstrated a significant improvement in both recruitment and activation of intra-tumour effector-type immune cells when SIRT was used in conjunction with pre-operative immunotherapy. A recently listed phase II prospective clinical trial is also under way to investigate the impact of SIRT with PD1-L and CTLA-4 inhibitors in subjects with intrahepatic biliary tract cancer (ClinicalTrials.gov Identifier: NCT04238637). Such data may prove to have a significant impact on the future management of ICC subjects receiving SIRT.

It may be that the issue of non-uniformity to lesion dosing is a barrier to ICC SIRT that cannot be easily overcome, however, the data from this study suggest that we may be able to deliver larger doses whilst remaining within safe limits to normal liver parenchyma. There was a mild correlation (*p* = 0.100) between average absorbed dose across healthy liver tissue and reduction in liver uptake rate, however even the largest cumulated dose to healthy liver across two consecutive SIRTs for a given subject (58 Gy) did not result in the subject moving from a healthy liver function status to a poor function status. Certainly subject history, standard liver function tests, and other comorbities must be taken into account, but such data do suggest we may be able to safely increase dosing to ICC subjects without inducing REILD, where clinical factors suggest it is safe to do so. Interestingly, Additional file 2: Figure S2 suggests subjects with a lower baseline function (or higher Bilirubin level) are not likely to experience a drop in function following SIRT, and may in fact experience an increase. This result is again in agreement with a previous study [17], and suggests a low liver uptake rate alone should not preclude HCC/ICC subjects from SIRT. However, it should be noted that the time of measurement of follow-up function could play a role in these measured trends and may be an area of future investigation. Ideally, the study would extend to include regional changes of function as measured by mebrofenin, such that local changes in function of spared and treated volumes could be compared. Unfortunately, this study did not include significant numbers of subjects undergoing selective (as opposed to whole liver) treatment and thus this analysis was not possible.

This study chose to use TLG as the metric of response, examining metabolic response to radiation absorbed dose as opposed to volume of disease. In some respects, reduction in volume of disease is a valuable outcome as that may be the primary intention of SIRT with a view to moving disease toward a resectable status. Long-term follow-up radiological exams were not available on the full cohort of subjects and it may be expected that such anatomical changes would not be evident at the time of early follow-up PET (~ 8 weeks post-SIRT). Correlation between the metabolic response demonstrated in this study with long-term survival benefits is an area of future work and, indeed, previous literature has demonstrated such a relationship when using the SIRT planning procedure ([^99m^Tc]MAA) to estimate tumour dose [5] as well as ^90^Y PET data [10].

## Conclusion

Treatment of ICC with SIRT is both safe and effective at stabilizing disease. The non-uniformity of dose across large lesions appears to be a critical factor in preventing a complete metabolic response even when high average dosing is achieved and as such EUD may be a more appropriate factor when predicting outcome. Baseline TLG may also be a critical factor when predicting response to SIRT. Typical absorbed dose to healthy liver is well tolerated and may indicate higher doses can be delivered in a safe approach to subject-specific treatment plans.

## Supplementary Information

**Additional file 1**. **Figure S1**. Display and analysis of ICC lesion: baseline [^18^F]FDG PET/CT (top row); ^90^Y derived dose map (middle row); and follow-up [^18^F]FDG PET/CT acquired 8 weeks post-SIRT (bottom row). The DVH for the lesion contour defined via FDG thresholding at baseline is shown on the right hand side. In this case, for a *D*_avg_ of 172 Gy, a stable metabolic response was achieved (reduction in TLG of 8%)

**Additional file 2**. **Figure S2**. Change in liver function, as measured by the global liver uptake rate (%/min) from ^99m^Tc-mebrofenin dynamic scintigraphy, with the baseline liver uptake rate. A positive change in uptake rate represents an improvement in liver function post-SIRT (*R*^2^ of linear fit is 0.199)

**Additional file 3**. **Figure S3**. EUD as a function of mean absorbed dose for the ICC cohort. Linear fit *y* = *0.62x*, R^2^ = 0.73. Solid line represents a linear relationship with slope of 1.

**Additional file 4**. **Table 4S.** Significance of parameters tested in the univariate binary logistic regression analysis when investigating prognostic factors for a significant reduction in normal liver uptake rate following SIRT.

## Data Availability

At this time the data analysed as part of this study is not publicly available.

## Abbreviations

ANOVA: Analysis of variance
BED: Biological effective dose
COV: Coefficient of variation
*D*_70_: Minimum dose to 70% of a volume
*D*_avg_: Average dose
DKC: Dose kernel convolution
DVH: Dose volume histogram
EBRT: External beam radiotherapy
FDG: Fluorodeoxyglucose
HCC: Hepatocellular carcinoma
ICC: Intrahepatic cholangiocarcinoma
MAA: Macroaggregated albumin
mCRC: Metastatic colorectal cancer
PET/CT: Positron emission tomography/X-ray computed tomography
REILD: Radioembolisation induced liver disease
SIRT: Selective internal radiation therapy
SUV: Standardised uptake value
TLG: Total lesion glycolysis
TcSC: [^99M^Tc]Sulphur colloid (TcSC)
*T*_eff_: Effective half life
*T*_rep_: Repair half time
*V*_50_: Volume receiving at least 50 Gy

## References

[1] Dhanasekaran R, Hemming AW, Zendejas I (2013). Treatment outcomes and prognostic factors of intrahepatic cholangiocarcinoma. Oncol Rep.

[2] Mouli S, Memon K, Baker T (2013). Yttrium-90 radioembolization for intrahepatic cholangiocarcinoma: safety, response, and survival analysis. J Vasc Intervent Radiol: JVIR.

[3] Zhen YA-O, Liu BA-O, Chang ZA-O, Ren HA-O, Liu ZA-OX, Zheng JA-O (2019). A pooled analysis of transarterial radioembolization with yttrium-90 microspheres for the treatment of unresectable intrahepatic cholangiocarcinoma. OncoTargets Therapy.

[4] Manceau V, Palard X, Rolland Y (2018). A MAA-based dosimetric study in patients with intrahepatic cholangiocarcinoma treated with a combination of chemotherapy and 90Y-loaded glass microsphere selective internal radiation therapy. Eur J Nucl Med Mol Imaging.

[5] Lam MGEH, Banerjee A, Goris ML (2015). Fusion dual-tracer SPECT-based hepatic dosimetry predicts outcome after radioembolization for a wide range of tumour cell types. Eur J Nucl Med Mol Imaging.

[6] Levillain HA-OX, Duran Derijckere I, Ameye L (2019). Personalised radioembolization improves outcomes in refractory intra-hepatic cholangiocarcinoma: a multicenter study. Eur J Nucl Med Mol Imaging.

[7] Wondergem M, Smits M, Elschot M (2013). Tc-99m-macroaggregated albumin poorly predicts the intrahepatic distribution of Y-90 resin microspheres in hepatic radioembolization. J Nucl Med: Off Publ Soc Nucl Med.

[8] Lhommel R, Elmbt L, Goffette P (2010). Feasibility of 90Y TOF PET-based dosimetry in liver metastasis therapy using SIR-spheres. Eur J Nucl Med Mol Imaging.

[9] Song YS, Paeng JC, Kim H-C (2015). PET/CT-based dosimetry in 90Y-microsphere selective internal radiation therapy: single cohort comparison with pretreatment planning on (99m)Tc-MAA imaging and correlation with treatment efficacy. Medicine (Baltimore).

[10] Willowson K, Hayes A, Bernard E, Maher R, Bailey D (2017). Image based prognostic factors in predicting response to Y-90 SIRT. J Nucl Med.

[11] Levillain H, Duran Derijckere I, Marin G (2018). (90)Y-PET/CT-based dosimetry after selective internal radiation therapy predicts outcome in patients with liver metastases from colorectal cancer. EJNMMI Res.

[12] Kafrouni M, Allimant C, Fourcade M (2019). Analysis of differences between 99mTc-MAA SPECT- and 90Y-microsphere PET-based dosimetry for hepatocellular carcinoma selective internal radiation therapy. EJNMMI Res.

[13] Levillain HA-OX, Bagni OA-O, Deroose CA-O (2021). International recommendations for personalised selective internal radiation therapy of primary and metastatic liver diseases with yttrium-90 resin microspheres. Eur J Nucl Med Mol Imaging.

[14] Willowson KP, Tapner M, Team Q, Bailey DL. A multicentre comparison of quantitative (90)Y PET/CT for dosimetric purposes after radioembolization with resin microspheres: the QUEST phantom study. Eur J Nucl Med Mol Imaging. 2015;42(8):1202–22. 10.1007/s00259-015-3059-9 10.1007/s00259-015-3059-9 PMC4480824 25967868

[15] Cremonesi M, Chiesa C, Strigari L (2014). Radioembolization of hepatic lesions from a radiobiology and dosimetric perspective. Fron Oncol.

[16] d’Abadie P, Hesse M, Jamar F, Lhommel R, Walrand S (2018). (90)Y TOF-PET based EUD reunifies patient survival prediction in resin and glass microspheres radioembolization of HCC tumours. Phys Med Biol.

[17] Willowson KP, Schembri GP, Bernard EJ, Chan DL, Bailey DL (2020). Quantifying the effects of absorbed dose from radioembolisation on healthy liver function with [99mTc]Tcmebrofenin. Eur J Nucl Med Mol Imaging.

[18] Tann M, Woolen S, Swallen A, Nelson A, Fletcher J (2015). Establishing a normal reference range for mebrofenin clearance rate (MCR) for overall liver function assessment. J Nucl Med.

[19] Dewaraja YK, Devasia T, Kaza RK (2020). Prediction of tumor control in 90Y radioembolization by logit models with PET/CT-based dose metrics. J Nucl Med.

[20] Tao R, Krishnan S, Bhosale PR (2016). Ablative radiotherapy doses lead to a substantial prolongation of survival in patients with inoperable intrahepatic cholangiocarcinoma: a retrospective dose response analysis. J Clin Oncol.

[21] Abbott EM, Falzone N, Lee BQ (2020). The impact of radiobiologically-informed dose prescription on the clinical benefit of yttrium-90 SIRT in colorectal cancer patients. J Nucl Med.

[22] Gholami Y, Willowson K, Forwood N (2018). Comparison of radiobiological parameters for 90Y radionuclide therapy (RNT) and external beam radiotherapy (EBRT) in vitro. EJNMMI Phys.

[23] Craciun L, de Wind R, Demetter P (2020). Retrospective analysis of the immunogenic effects of intra-arterial locoregional therapies in hepatocellular carcinoma: a rationale for combining selective internal radiation therapy (SIRT) and immunotherapy. BMC Cancer.

