# Supplementary figures and images for: Individualised dosimetry and safety of SIRT for intrahepatic cholangiocarcinoma
Source: EJNMMI Phys. 2021 Sep 14;8:65. doi: 10.1186/s40658-021-00406-2 (PMC8440713; doi:10.1186/s40658-021-00406-2)

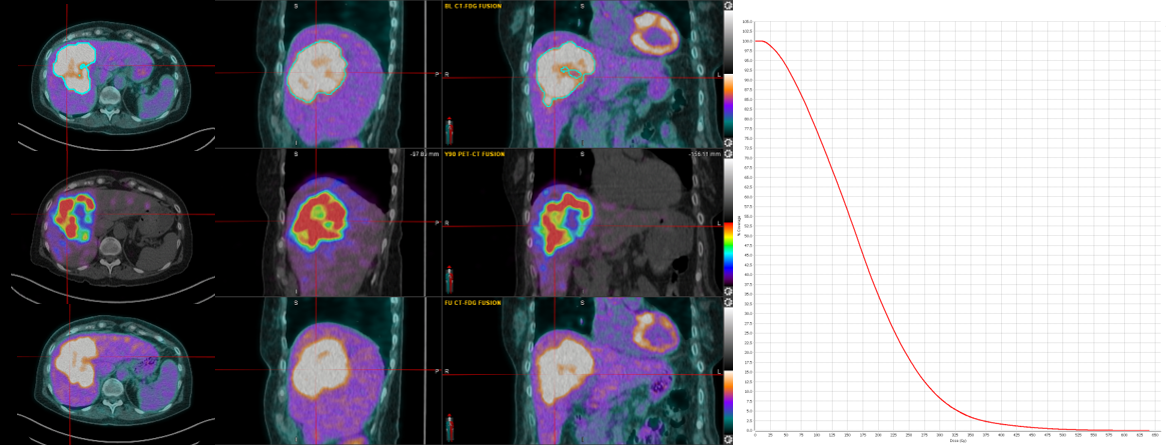

Supplement: Supplementary file 1 — Additional file 1. Figure S1. Display and analysis of ICC lesion: baseline [18F]FDG PET/CT (top row); 90Y derived dose map (middle row); and follow-up [18F]FDG PET/CT acquired 8 weeks post-SIRT (bottom row). The DVH for the lesion contour defined via FDG thresholding at baseline is shown on the right hand side. In this case, for a Davg of 172 Gy, a stable metabolic response was achieved (reduction in TLG of 8%) [file 40658_2021_406_MOESM1_ESM.png]

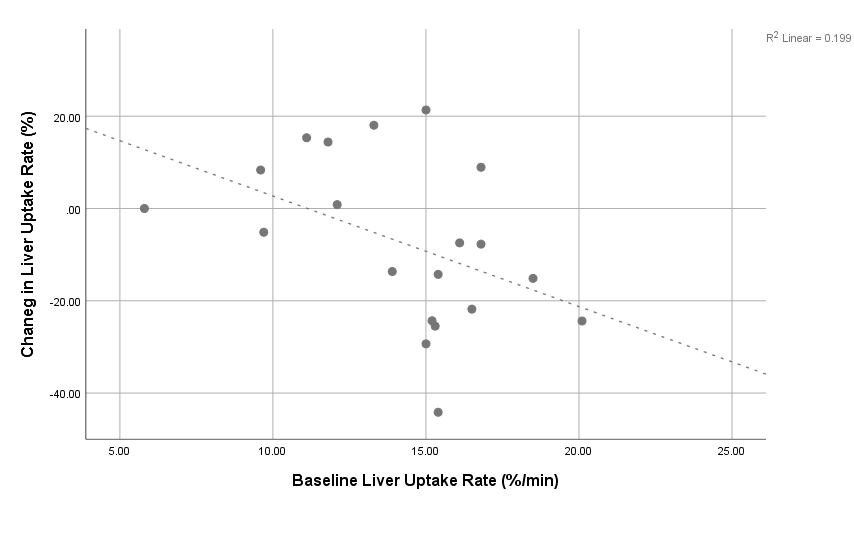

Supplement: Supplementary file 2 — Additional file 2. Figure S2. Change in liver function, as measured by the global liver uptake rate (%/min) from 99mTc-mebrofenin dynamic scintigraphy, with the baseline liver uptake rate. A positive change in uptake rate represents an improvement in liver function post-SIRT (R2 of linear fit is 0.199) [file 40658_2021_406_MOESM2_ESM.png]

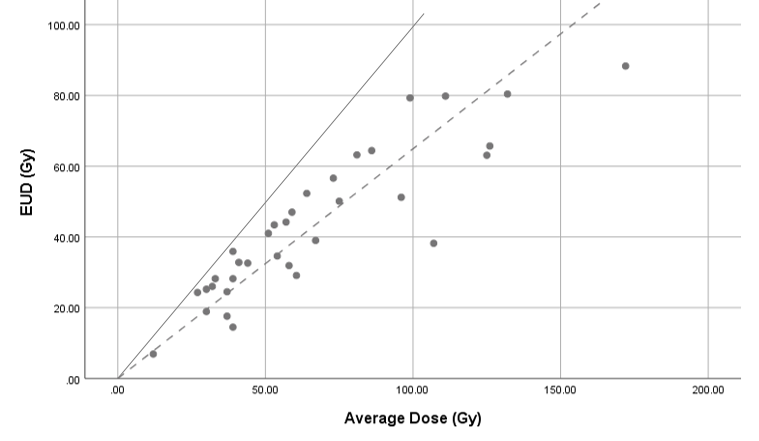

Supplement: Supplementary file 3 — Additional file 3. Figure S3. EUD as a function of mean absorbed dose for the ICC cohort. Linear fit y = 0.62x, R2 = 0.73. Solid line represents a linear relationship with slope of 1. [file 40658_2021_406_MOESM3_ESM.png]
