# Supplementary material for: Individualised dosimetry and safety of SIRT for intrahepatic cholangiocarcinoma
Source: EJNMMI Phys. 2021 Sep 14;8:65. doi: 10.1186/s40658-021-00406-2 (PMC8440713; doi:10.1186/s40658-021-00406-2)
Supplement: Supplementary file 4 — Additional file 4. Table 4S. Significance of parameters tested in the univariate binary logistic regression analysis when investigating prognostic factors for a significant reduction in normal liver uptake rate following SIRT. [file 40658_2021_406_MOESM4_ESM.docx]

Table 4S: significance of parameters tested in the univariate binary logistic regression analysis when investigating prognostic factors for a significant reduction in normal liver uptake rate following SIRT.

| **Parameter** | **p-value** |
| --- | --- |
| D_avg_^*^ | 0.155 |
| Baseline Bilirubin | 0.445 |
| Baseline liver uptake rate | 0.098 |
| Tumour burden | 0.181 |

*^*^Average dose*
